# AI in Home Care—Evaluation of Large Language Models for Future Training of Informal Caregivers: Observational Comparative Case Study

**DOI:** 10.2196/70703

**Published:** 2025-04-28

**Authors:** Clara Pérez-Esteve, Mercedes Guilabert, Valerie Matarredona, Einav Srulovici, Susanna Tella, Reinhard Strametz, José Joaquín Mira

**Affiliations:** 1 Fundación para el Fomento de la Investigación Sanitaria y Biomédica de la Comunitat Valenciana Centro de Salud Hospital-Plá Alicante Spain; 2 Health Psychology Department Miguel Hernandez University Elche Spain; 3 Fundación para el Fomento de la Investigación Sanitaria y Biomédica de la Comunitat Valenciana Alicante Spain; 4 Department of Nursing University of Haifa Haifa Israel; 5 Health and Wellbeing Department LAB University of Applied Sciences Lappeenranta Finland; 6 Wiesbaden Institute for Healthcare Economics and Patient Safety RheinMain University of Applied Sciences Wiesbaden Germany

**Keywords:** large language models, older adults, informal caregiver, error prevention, patient safety, ChatGPT, Microsoft Copilot, training, health literacy

## Abstract

**Background:**

The aging population presents an accomplishment for society but also poses significant challenges for governments, health care systems, and caregivers. Elevated rates of functional limitations among older adults, primarily caused by chronic conditions, necessitate adequate and safe care, including in-home settings. Traditionally, informal caregiver training has relied on verbal and written instructions. However, the advent of digital resources has introduced videos and interactive platforms, offering more accessible and effective training. Large language models (LLMs) have emerged as potential tools for personalized information delivery. While LLMs exhibit the capacity to mimic clinical reasoning and support decision-making, their potential to serve as alternatives to evidence-based professional instruction remains unexplored.

**Objective:**

We aimed to evaluate the appropriateness of home care instructions generated by LLMs (including GPTs) in comparison to a professional gold standard. Furthermore, it seeks to identify specific domains where LLMs show the most promise and where improvements are necessary to optimize their reliability for caregiver training.

**Methods:**

An observational, comparative case study evaluated 3 LLMs—GPT-3.5, GPT-4o, and Microsoft Copilot—in 10 home care scenarios. A rubric assessed the models against a reference standard (gold standard) created by health care professionals. Independent reviewers evaluated variables including specificity, clarity, and self-efficacy. In addition to comparing each LLM to the gold standard, the models were also compared against each other across all study domains to identify relative strengths and weaknesses. Statistical analyses compared LLMs performance to the gold standard to ensure consistency and validity, as well as to analyze differences between LLMs across all evaluated domains.

**Results:**

The study revealed that while no LLM achieved the precision of the professional gold standard, GPT-4o outperformed GPT-3.5, and Copilot in specificity (4.6 vs 3.7 and 3.6), clarity (4.8 vs 4.1 and 3.9), and self-efficacy (4.6 vs 3.8 and 3.4). However, the models exhibited significant limitations, with GPT-4o and Copilot omitting relevant details in 60% (6/10) of the cases, and GPT-3.5 doing so in 80% (8/10). When compared to the gold standard, only 10% (2/20) of GPT-4o responses were rated as equally specific, 20% (4/20) included comparable practical advice, and just 5% (1/20) provided a justification as detailed as professional guidance. Furthermore, error frequency did not differ significantly across models (*P*=.65), though Copilot had the highest rate of incorrect information (20%, 2/10 vs 10%, 1/10 for GPT-4o and 0%, 0/0 for GPT-3.5).

**Conclusions:**

LLMs, particularly GPT-4o subscription-based, show potential as tools for training informal caregivers by providing tailored guidance and reducing errors. Although not yet surpassing professional instruction quality, these models offer a flexible and accessible alternative that could enhance home safety and care quality. Further research is necessary to address limitations and optimize their performance. Future implementation of LLMs may alleviate health care system burdens by reducing common caregiver errors.

## Introduction

### Home Care

An aging population represents a significant achievement for society, but it also presents challenges for governments, health agencies, social institutions, health care providers, and informal caregivers [1]. The accumulation of health risks over the life course, including diseases, injuries, and chronic conditions, contributes to higher disability rates among older adults, highlighting the need for specialized care, including home-based care. These demands are projected to increase in the coming decades [2].

A key strategy to address this challenge involves ensuring that care provided at home for dependent individuals by family members or hired informal caregivers is both adequate and safe. Enhancing the safety of home care for chronically ill individuals is critical within the framework of the emerging care economy [3].

Traditionally, caregiver training has relied on verbal and written instructions. However, with the proliferation of digital resources, tools such as videos, blogs, and websites that integrate text and images interactively have become widespread due to their ability to reach larger audiences at lower costs. Furthermore, innovative methods such as immersive audiovisual materials and virtual reality are being explored to train informal caregivers [4].

In this context, technological advancements, particularly in large language models (LLMs), are increasingly being explored as tools to enhance caregiver education. These artificial intelligence (AI)–driven models, powered by generative AI, provide new ways to deliver personalized, interactive learning experiences that can complement and expand upon traditional training methods, offering significant potential to improve the effectiveness and accessibility of caregiver support and training [5].

### LLMs

The recent emergence of generative AI models referred to as LLMs, has introduced new opportunities in AI [5]. Currently, various LLM applications are accessible to the public, either free of charge or through subscription services [4]. These applications promise to deliver personalized, cost-effective information, positioning themselves as versatile tools for educating informal caregivers [6].

GPTs represent a major breakthrough in AI, forming a family of LLMs built on a deep learning architecture known as transformers [7]. Developed by OpenAI, these models serve as the foundational technology behind ChatGPT and other generative AI applications, enabling them to produce content that remarkably resembles human-created outputs. While their capabilities are already impressive, their practical applications are being gradually explored and expanded, unlocking new possibilities across various fields [8].

Although GPTs and LLMs have limitations, they demonstrate capabilities such as mimicking clinical reasoning, interpreting images or other diagnostic results, synthesizing patient information, and facilitating clinical decision-making [5]. Their applications extend to both research and medical practice [9]. For example, a recent study demonstrated their utility in migraine education, where LLMs were found effective in offering advice, improving information comprehension, and providing personalized responses for non–health care professionals [5].

Despite their growing popularity and daily use, it seems unlikely that LLMs can serve as viable alternatives to evidence-based or consensus-based instructions provided by professionals for training informal caregivers [10].

### Objectives

This study aims to evaluate the appropriateness of home care instructions generated by LLMs (including GPTs) in comparison to a professional gold standard. It investigates whether LLM-generated recommendations can be both reliable and useful for supporting patient care in nonprofessional home settings. Specifically, it examines how informal caregivers, seeking to deliver safer and more appropriate home care, can use simple prompts to retrieve accurate information from LLMs. As secondary objectives, the study compares the outputs of different LLM systems and identifies errors arising from interactions with these models.

## Methods

### Design

An observational, comparative case study was conducted from July to November 2024, following these 5 steps mentioned in Textbox 1.

**Figure 1:**
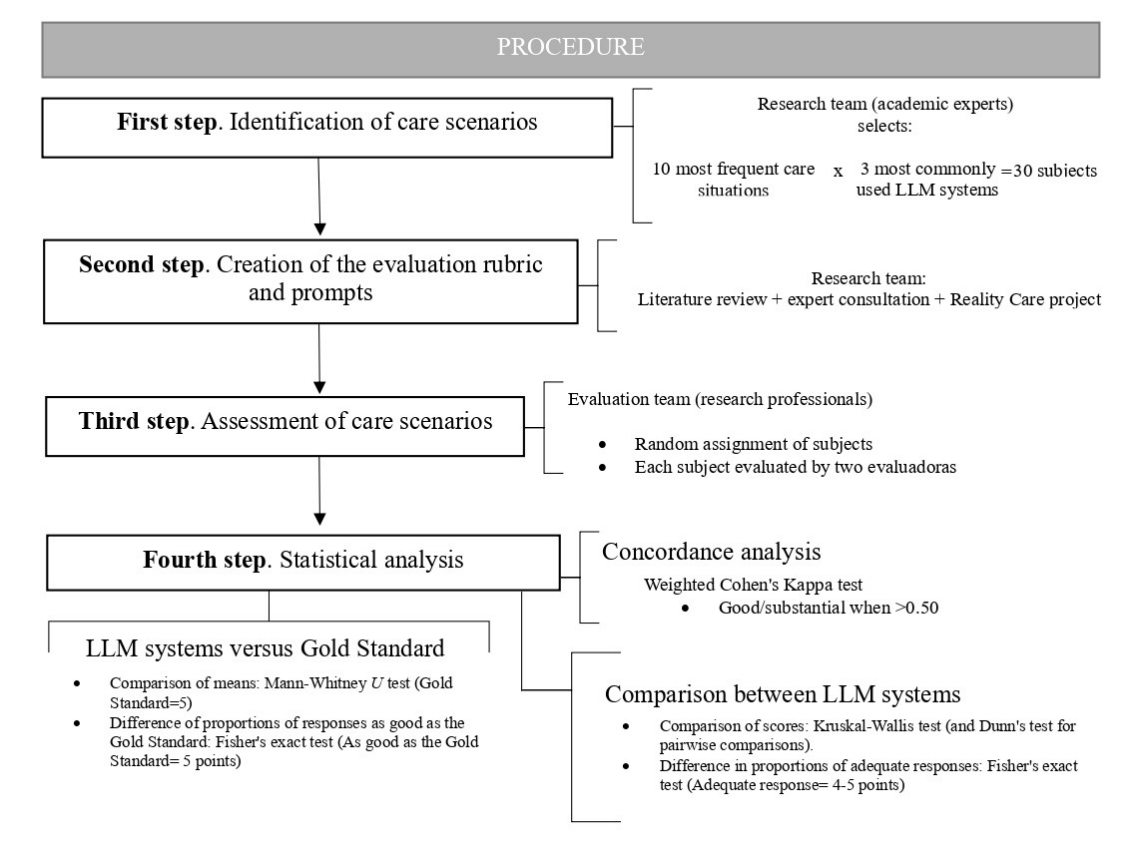
Study flowchart. LLM: large language model.

Refer to Figure 1 for the study flowchart.

**Five-step methodology for evaluating large language models' performance in home care scenarios.**

- Selection of scenarios: 10 common and frequent home care situations involving dependent older patients managed by informal caregivers were identified.
- Development of a rubric: a standardized rubric was created to assess all scenarios, with specific modifications tailored to individual situations. In parallel, a panel of 18 health care professionals (research team) developed detailed, evidence-based instructions (gold standard) to guide informal caregivers in performing home care tasks.
- Prompt generation: for each care scenario, specific prompts were used to gather responses from 2 versions of GPT (one free and one paid) and Copilot.
- Statistical analysis: the responses were analyzed statistically to evaluate interrater agreement and ensure consistency in scoring.
- Performance comparison: The outputs of the different large language models were compared against each other and the gold standard using the rubric by the evaluation team. A concordance analysis among researchers further validated the reliability of evaluations.

### First Step: Identification of Care Scenarios

#### Selection of Care Scenarios

The research team identified common home care situations through a qualitative approach, incorporating insights from a large cohort of nursing professionals in primary care and home hospital care settings. These scenarios were highlighted as part of the Reality Care Project [11], which also aims to compare various educational strategies for training informal caregivers to reduce medication errors and improve home care safety across diverse settings.

In total, 10 common and critical scenarios (Table 1) were subsequently selected. These scenarios represent situations frequently encountered by informal caregivers providing home-based care to older adults, including those with disabilities, chronic or terminal illnesses, or undergoing rehabilitation, who require assistance with daily activities. These are everyday situations in which an informal caregiver looks after an individual who may experience one or more cardiometabolic conditions, cancer, Alzheimer disease, or other chronic illnesses.

These scenarios encompass tasks such as operating medical devices, assisting with mobility, performing wound care, and managing medication—responsibilities essential for maintaining the patient’s health and well-being at home. The selection criteria were based on the scenarios' prevalence, complexity, and associated risks of improper management. These scenarios cover a range of essential care tasks, including both routine and emergency interventions, emphasizing the importance of accurate instruction and guidance to safeguard patient safety and well-being.

**Table 1:** Selected home care scenarios.

Scenario	Description
Insulin administration	Managing diabetes through regular insulin injections, which require precise dosing and timing to avoid serious health complications.
Porth-a-cath maintenance	Caring for central venous access devices, necessitates sterile handling to prevent infections.
Diaper changing	A routine but essential task for maintaining patient hygiene, and preventing skin issues, particularly for patients with limited mobility.
Patient transfers	Safe transfer techniques to reduce the risk of falls and physical strain on both patients and caregivers.
Heart failure management	Recognizing and responding to symptoms of heart failure to prevent exacerbations that could require emergency care.
Blood pressure monitoring	Routine blood pressure checks to manage hypertension and detect changes that may indicate health deterioration.
Choking maneuvers	Knowledge of emergency techniques to respond to airway obstructions, a common risk among elderly patients.
Caregiver self-care	Techniques to prevent burnout and maintain the caregiver’s physical and mental health, as they play a crucial role in patient care.
Pressure ulcer prevention	Skincare and repositioning strategies to prevent pressure sores in bedridden patients.
Hand hygiene	Fundamental practices for infection prevention, critical for any caregiving scenario.

#### Selection of LLM

The research team selected the LLM systems for the study based on their popularity and demonstrated performance in previous research. Three models were included, each representing widely adopted and validated alternatives with varying capabilities, which are mentioned below in Textbox 2.

**Large language models were selected for comparative evaluation based on popularity and previous performance.**

- GPT-3.5 (released in 2020), is a free version of GPT capable of generating human-like text, understanding nuanced contexts, and performing complex linguistic tasks.
- GPT-4o (launched in 2023), a paid version of GPT with enhanced performance, accuracy, and contextual understanding, is designed to handle sophisticated linguistic tasks and generate coherent, contextually relevant responses.
- Copilot (introduced in June 2021), is designed to assist developers by providing real-time code suggestions, auto-completion, and full code snippets.

This selection ensures a comprehensive evaluation by considering these widely used models, which have been validated in numerous studies and applications, ensuring both general and specialized relevance.

#### Gold Standard

From the Reality Care Project, detailed scripts for the 10 selected scenarios were used to establish the gold standard. These scripts described each step necessary for executing care tasks effectively and safely. Each protocol included step-by-step instructions with critical information explaining the rationale behind each action. Specific prompts and contextual examples were incorporated to guide caregivers in adapting their approaches as needed. These scripts reflected consensus among professionals, offering a structured and comprehensive reference for best practices in caregiving. Their validity was rigorously ensured in earlier phases of the Reality Care Project through a structured process, where each script was independently reviewed by 2 experts from different centers, each with at least five years of experience in the field.

### Second Step: Creation of the Evaluation Rubric and Prompts

#### Rubric Development

A rubric based on a review of scientific literature, expert consultations, and insights from the Reality Care Project was developed. The rubric was designed to evaluate LLM performance across caregiving scenarios and to compare LLM-generated responses to the gold standard (refer to Multimedia Appendix 1).

The rubric comprised 2 main sections (Textbox 3).

Each item was scored on a Likert scale from 1 (lowest) to 5 (highest). In addition, qualitative data on errors were collected, with specific examples provided to illustrate common deviations from the gold standard.

**Evaluation rubric for assessing responses of large language models (LLMs) and alignment with the gold standard in home care scenarios.**

**LLM performance evaluation**

- Specificity: level of detail provided relative to the scenario.
- Clarity: ease of understanding and practical application.
- Adaptability: ability to adjust responses to varying circumstances.
- Self-efficacy: confidence inspired in caregivers after reading the response.
- Error frequency: number of errors in the explanation.

**Alignment with the gold standard**

- Specificity: comparison of depth of detail with the gold standard.
- Practical advice: inclusion of actionable recommendations.
- Justification: explanation of why each action is necessary.
- Content: alignment with procedural steps from the gold standard.
- Overall evaluation: holistic comparison of the response to the gold standard.

#### Creation of Prompts

A set of 10 standardized prompts was developed to simulate the caregiving scenarios used in the study (refer to Multimedia Appendix 2). These prompts were specifically designed to generate clear and detailed instructions for informal caregivers lacking formal medical training. They emphasized step-by-step explanations to ensure caregiving practices were both safe and comprehensible. The same prompts were applied to all three LLMs, allowing for direct performance comparisons.

### Third Step: Assessment of Care Scenarios

The evaluation phase involved a team of reviewers (an evaluation team composed of MGM, VMP, AM, and JJM). Instructions were standardized to ensure uniformity in the assessment process. An initial concordance analysis was performed, achieving a 60% agreement threshold before the evaluation of all scenarios commenced.

Care scenarios and LLM systems were randomly and blindly assigned to reviewers. Each reviewer independently generated responses using the assigned prompts and scored each rubric variable on a scale of 1 to 5. Additional follow-up questions were occasionally posed to clarify or obtain further details (eg, “Please, provide more information on avoiding common errors in insulin administration”).

To enhance objectivity, each scenario was evaluated by 2 reviewers. This dual-review process minimized bias and ensured that the evaluations accurately reflected LLM performance relative to the gold standard. Qualitative feedback was also collected, documenting common types of errors and providing illustrative examples.

### Fourth Step: Statistical Analysis

#### Concordance Analysis Between Reviewers

CPE conducted a concordance analysis using the weighted version of the Cohen κ test to calculate agreement levels between reviewer pairs. Substantial agreement levels (62%-81%) were achieved, confirming consistency across evaluations.

#### Comparison Between LLMs

The performance of the 3 LLMs was compared across evaluation domains (specificity, clarity, adaptability, self-efficacy, and error frequency). A nonparametric Kruskal-Wallis test was used due to the nonnormal distribution of variables. The Dunn test with Bonferroni correction was used for post hoc pairwise comparisons. Items with scores of 4 or 5 were classified as “adequate responses,” and the Fisher exact test compared proportions of adequate responses across LLMs.

#### Comparison of LLMs Versus Gold Standard

LLMs’ scores were compared to those of the gold standard, which represented the maximum possible score of 5 points. Mann-Whitney *U* tests assessed differences in domain-specific performance, with a threshold of statistical significance at a *P* value of less than .05 (*P*≤.025 for the Dunn test). Items scoring 5 were categorized as “equally good as the gold standard,” while lower scores indicated deviations.

#### Identification of Errors in Interaction With LLM Systems

Common errors in LLM responses were classified into 5 categories (Textbox 4).

**Categories of errors observed in the outputs of large language models when responding to informal caregiving tasks.**

- Omitted relevant information: key details missing despite being within the model’s knowledge.
- Excessive information: the inclusion of irrelevant or redundant details, confuses.
- Misinterpreted queries: incorrect understanding of the question, leading to incoherent or irrelevant answers.
- Repetitive responses: identical or nearly identical responses to different questions.
- Incorrect information: responses containing factual inaccuracies or contradictions.

### Ethical Considerations

This study did not require new approval from the ethics committee, as the study procedures and materials were reviewed and approved in a previous phase of the Reality Care project. Ethical approval for that earlier phase was granted by the Research Ethics Committee of the San Juan University Hospital of Alicante in December 2021 and January 2023 (CODES 21/063; 22/079; and 22/080), and the protocol for that phase has been registered in ClinicalTrials.gov (NCT05885334).

## Results

### Description

In total, 60 responses were generated by the LLM systems (20 each from GPT-3.5, GPT-4o, and Copilot), with 6 responses corresponding to each of the 10 selected care scenarios (2 per LLM system).

### Interrater Agreement Results

Agreement levels among reviewers were substantial, ranging from 62% (7/11) to 81% (9/11). All results were statistically significant, confirming consistent evaluations across variables and scenarios (Table 2).

**Table 2:** Estimating agreement between each pair of assessors.

			Reviewer 1		Reviewer 2		Reviewer 3		Reviewer 4
**Reviewer 1**									
	Weighted Cohen κ	1.00		0.62		0.64		0.78	
	*P* value^a^	—^b^		.03		.03		<.01	
**Reviewer 2**									
	Weighted Cohen κ	0.62		1.00		0.73		0.81	
	*P* value	.03		—		<.01		.01	
**Reviewer 3**									
	Weighted Cohen κ	0.64		0.73		1.00		0.67	
	*P* value	.03		<.01		—		.02	
**Reviewer 4**									
	Weighted Cohen κ	0.78		0.81		0.67		1.00	
	*P* value	<.01		.01		.02		—	

^a^*P*<.05 indicates significant interrater agreement.

^b^Not applicable.

#### Comparison Between LLM Systems

Significant differences were observed between the LLMs across all study domains, except for error frequency (Table 3).

GPT-4o achieved a significantly higher specificity score (4.6 points) than both GPT-3.5 (3.7 points, *P*<.01) and Copilot (3.6 points, *P*<.01). In terms of clarity, GPT-4o also demonstrated superior performance (4.8 points) relative to GPT-3.5 (4.1 points, *P*<.01) and Copilot (3.9 points, *P*<.01). Regarding adaptability to variability, GPT-4o outperformed GPT-3.5 with a score of 2.5 versus 1.4 points (*P*<.01); however, no statistically significant difference was observed between GPT-4o and Copilot in this domain. Finally, GPT-4o showed higher performance in fostering caregiver self-efficacy (4.6 points), exceeding the scores of both GPT-3.5 (3.8 points, *P*=.02) and Copilot (3.4 points, *P*<.01).

No significant differences were identified between GPT-3.5 and Copilot across any of the evaluated domains.

The frequency of appropriate responses provided by each LLM across various domains is summarized in Table 4. Significant differences were identified in the following areas: (1) Specificity: GPT-4o demonstrated a significantly higher proportion of appropriate responses (19/20, 95%) compared to Copilot (10/20, 50%; *P*<.01); (2) Self-efficacy: GPT-4o also outperformed Copilot in this domain, with 90% (18/20) of responses deemed appropriate compared to 40% (8/20) for Copilot (*P*<.01).

For the remaining domains, no significant differences were observed in the proportion of appropriate responses among the LLMs.

**Table 3:** Scores achieved by each large language model across the different study domains.

	GPT-3.5	GPT-4o	Copilot	Chi-square (*df*)	*P* value^a^
Specificity	3.7	4.6	3.6	15.14 (2)	<0.01
Clarity	4.1	4.8	3.9	16.93 (2)	<0.01
Variability	1.4	2.5	2.0	8.66 (2)	0.01
Self-efficacy	3.8	4.6	3.4	15.26 (2)	<0.01
Error frequency	4.2	4.3	4.2	0.86 (2)	0.65

^a^Kruskal-Wallis nonparametric test. *P*<.05 indicates significant differences between the large language models.

**Table 4:** Frequency of appropriate responses provided by each LLM^a^.

Domain and LLM			Adequate response^b^, n (%)		GPT-4o versus GPT-3.5, *P*^b^ value		GPT-4o versus Copilot, *P*^c^ value		GPT-3.5 versus Copilot, *P*^c^ value
**Specificity**					.09		<.001		.33
	GPT-3.5	14 (70)							
	GPT-4o	19 (95)							
	Copilot	10 (50)							
**Clarity**					1.0		.10		.10
	GPT-3.5	20 (100)							
	GPT-4o	20 (100)							
	Copilot	16 (80)							
**Variability**					.10		.10		1.0
	GPT-3.5	0 (0)							
	GPT-4o	4 (20)							
	Copilot	0 (0)							
**Self-efficacy**					.40		<.001		.05
	GPT-3.5	15 (75)							
	GPT-4o	18 (90)							
	Copilot	8 (40)							
**Error frequency**					.23		1.0		.60
	GPT-3.5	17 (85)							
	GPT-4o	20 (100)							
	Copilot	19 (95)							

^a^LLM: large language model.

^b^To calculate the proportion of appropriate responses, we consider the evaluations provided by the two reviewers for each item.

^c^Fisher Exact test. *P*<.05 indicates significant differences between the proportion of adequate responses provided by each tool.

#### Comparison of LLMs Versus Gold Standard

Across all evaluated variables, the differences between the gold standard’s scores and those of the LLMs were statistically significant, confirming that none of the LLMs matched the performance of the gold standard (Table 5).

**Table 5:** Comparison of scores of the different LLMs^a^ with the gold standard.

	Gold standard	GPT-3.5			GPT-4o			Copilot	
	Mean	Mean (SD)	*P* value^b^	Mean (SD)		*P* value^b^	Mean (SD)		*P* value^b^
Specificity	5.0	2.9 (1.0)	<.01	3.3 (1.0)		<.01	2.7 (1.1)		<.01
Practical advice	5.0	2.5 (1.0)	<.01	3.9 (0.7)		<.01	2.8 (0.8)		<.01
Justification	5.0	2.1 (0.9)	<.01	3.5 (0.6)		<.01	2.2 (1.0)		<.01
Content	5.0	2.7 (0.6)	<.01	3.0 (0.9)		<.01	3.2 (0.7)		<.01
Overall evaluation	5.0	2.6 (0.7)	<.01	3.1 (1.0)		<.01	2.5 (0.7)		<.01

^a^LLM: large language model.

^b^Mann-Whitney *U* test. *P*<.05 indicates significant differences between the score obtained by the LLM and the gold standard, where we assume the maximum score.

Nevertheless, GPT-4o consistently achieved the highest scores among the LLMs, outperforming GPT-3.5 across all variables and surpassing Copilot in all but the content domain. In the content domain, Copilot scored slightly higher (3.2 points) compared to GPT-4o (3.0 points).

#### Specificity and Alignment With the Gold Standard

In terms of specificity, none of the responses from GPT-3.5 were considered as specific or detailed as the gold standard (Table 6). However, 1 response from Copilot (1/20, 5%) and 2 responses from GPT-4o (2/20, 10%) were deemed comparable to the gold standard.

**Table 6:** Classification of the responses generated by LLMs^a^ compared to the gold standard.

Domain and accuracy^b^		GPT-3.5	GPT-4o	Copilot
**Specificity, n (%)**				
	As good as gold standard	0 (0)	2 (10)	1 (5)
	Worse than gold standard	20 (100)	18 (90)	19 (95)
**Practical advice, n (%)**				
	As good as gold Standard	0 (0)	4 (20)	0 (0)
	Worse than gold standard	20 (100)	16 (80)	20 (100)
**Justification, n (%)**				
	As good as gold standard	0 (0)	1 (5)	0 (0)
	Worse than gold standard	20 (100)	19 (95)	20 (100)
**Content, n (%)**				
	As good as gold standard	0 (0)	0 (0)	0 (0)
	Worse than gold standard	20 (100)	20 (100)	20 (100)
**Overall evaluation, n (%)**				
	As good as gold standard	0 (0)	0 (0)	0 (0)
	Worse than gold standard	20 (100)	20 (100)	20 (100)

^a^LLM: large language model.

^b^To calculate the proportion of appropriate responses we consider the evaluations provided by the 2 reviewers for each item.

For practical advice -such as reminders or recommendations (eg, “Remember that the first aid kit should be out of reach of children or the elderly”), none of the responses from GPT-3.5 or Copilot matched the gold standard, while 4 responses (4/20, 20%) from GPT-4o were rated as ideal.

Regarding justification of actions, which involves explaining why specific actions are necessary (eg, in the “Port-a-Cath” scenario, prompting for signs of redness, blackness, or purple discoloration at the insertion site), no responses from GPT-3.5 or Copilot met the gold standard. Only 1 response (1/20, 5%) from GPT-4o was considered ideal.

In terms of content, which assesses whether all necessary procedural steps were included, none of the responses from GPT-3.5, GPT-4o, or Copilot were considered as comprehensive as the gold standard.

Overall, explanations generated by all LLMs were rated as inferior to those provided by the gold standard.

#### Error Analysis

No significant differences were observed between GPT-3.5, GPT-4o, and Copilot in the types of errors they most frequently made (*P*=.88). However, specific patterns emerged (Textbox 5).

Although variations in error patterns were noted, none of these differences reached statistical significance across the models (Table 7).

**Common error types in large language models’ outputs: specific trends observed across models.**

- Omission of relevant information: GPT-3.5 was the most likely to omit critical details, doing so in 80% (8/10) of caregiving scenarios (eg, failing to explain how to avoid injury to the caregiver during patient transfers), compared to 60% (6/10) for both GPT-4o and Copilot.
- Providing excessive information: Both GPT-3.5 and Copilot provided more information than necessary in 40% (4/10) of cases, whereas GPT-4o did so in only 20% (2/10).
- Failure to understand the question: GPT-3.5 repeated irrelevant explanations (eg, reiterating the insulin administration process instead of addressing errors) in 20% (2/10) of cases. GPT-4o and Copilot demonstrated this issue in 10% (1/10) of cases each.
- Repetition of responses: Both GPT-3.5 and GPT-4o repeated identical or nearly identical answers to different questions in 30% (3/10) of scenarios, compared to only 10% (1/10) for Copilot.
- Providing incorrect information: GPT-3.5 did not provide incorrect information in any scenario. In contrast, GPT-4o did so in 10% (1/10) of cases, and Copilot in 20% (2/10).

**Table 7:** Types of errors made by each large language model. Fisher Exact Test (*P*=.87).

Large language models and categories		Errors, n (%)
**GPT-3.5**		
	Omitted relevant information	8 (80)
	Provided more information than necessary	4 (40)
	Did not understand the question	2 (20)
	Repeated the same response to different questions	3 (30)
	Provided incorrect information	0 (0)
**GPT-4o**		
	Omitted relevant information	6 (60)
	Provided more information than necessary	2 (20)
	Did not understand the question	1 (10)
	Repeated the same response to different questions	3 (30)
	Provided incorrect information	1 (10)
**Copilot**		
	Omitted relevant information	6 (60)
	Provided more information than necessary	4 (40)
	Did not understand the question	1 (10)
	Repeated the same response to different questions	1 (10)
	Provided incorrect information	2 (20)

## Discussion

### Principal Findings

The number of older adults living at home and requiring care is increasing rapidly, and in parallel, so is the number of informal caregivers who must acquire diverse competencies at different stages of the care recipient’s condition [12]. Most frequently, informal caregivers need guidance on hygiene, nutrition, and caregiver respite, areas consistently identified as their main concerns [13]. Given widespread internet access, caregivers commonly seek information online, leading to the development of numerous dedicated websites and digital resources [14]. Over the years, the quality and reliability of these sources have improved [15,16]. Now, with the emergence of new AI technologies, particularly LLMs, novel opportunities for caregiver support are arising opportunities that warrant careful exploration and evaluation.

### Findings

The information provided by LLMs is currently acceptable; however, the insights offered by expert professionals remain more comprehensive. Notably, GPT-4o generated instructions that closely aligned with the gold standard. This study confirms that AI-based technologies in medicine are advancing rapidly and are beginning to see real-world clinical implementation. However, as others have noted, further enhancements are still necessary [17].

The introduction of LLMs in training informal caregivers offers several advantages over traditional methods [8]. LLMs are accessible and provide immediate, tailored responses to users' specific situations [5]. This is particularly beneficial for informal caregivers, who often lack previous training and require clear, concise guidance in real time to avoid mistakes [7]. These findings highlight the urgency of developing guidelines for prompt design and ensuring the safe use of LLMs by informal caregivers. Given the rapid dissemination and advanced capabilities of these systems, addressing this issue is critical.

### AI for Informal Caregiver Support

AI has been applied in other fields to support informal caregivers, including assessing emotional burdens and providing support through chatbots. While these applications have shown acceptable results, AI remains underexplored as a tool for enhancing caregiver training or offering guidance on safe care practices.

In addition, AI has demonstrated potential in decision-making support through trained algorithms, integrating data from home sensors to enable improved remote monitoring [18]. Furthermore, AI contributes to better coordination between formal and informal caregivers, paving the way for more advanced telehealth solutions [19]. However, the use of LLMs to provide informal caregivers with personalized, accurate information for safe care has been minimally studied. The impact of LLMs on patient safety is nonetheless critical.

### Integrative Insights

This study highlights the ability of GPT-4o to adapt to contextual needs and provide accurate, detailed instructions, enhancing the safety of home care. Previous studies [5,20] have also reported that GPT-4o outperforms other LLMs [5,7,8]. In this study, GPT-4o surpassed GPT-3.5 and Copilot in key variables such as specificity, clarity, and self-efficacy. These characteristics are essential for informal caregivers, who often perform high-risk tasks such as medication administration without direct professional supervision. However, the fact that GPT-4o can provide answers with greater clarity and relevance contributes to fostering a sense of security among caregivers which could lead to unintentional errors.

The comparison of LLMs with the gold standard revealed that while none achieved the accuracy and detail of professional instructions, these models provided adequate responses in many scenarios [21]. For instance, LLMs offered sufficient guidance for insulin administration and choking maneuvers, enabling caregivers to perform these tasks safely. However, professional intervention remains necessary in certain cases to ensure quality care, as LLMs can omit details or oversimplify critical steps [10].

Unlike concerns reported in other AI applications, hallucinations—fabrications or inventions—were not observed in this study. However, errors and disinformation are typically described when using LLMs in the health setting [22].

LLMs also offer advantages over traditional written or audiovisual training by enabling dynamic and adaptive interaction based on caregivers' questions [23]. Caregivers can ask additional questions to clarify instructions or request helpful visual aids, improving understanding and confidence [17]. The variability and adaptability of GPT-4o were particularly notable, offering greater personalization than traditional models [24]. This is particularly relevant in cases involving caregivers from migrant backgrounds, for whom language and cultural differences may pose barriers, that LLMs can help to overcome by offering clear, accessible, and culturally adaptable information [25]. Future developments, such as AI-powered video generation to illustrate tasks, could further enhance these systems' utility [26].

To maximize their effectiveness, informal caregivers must actively generate appropriate prompts to ensure informed decision-making based on LLM-provided information. As clinical guidelines for AI implementation exist, it is vital to establish similar frameworks for informal caregivers [27].

LLMs offer information that can be tailored to better understand how to provide care to a dependent patient at home. This aspect still needs to be further explored. By considering potential risks in each situation, these systems can help caregivers minimize errors and prevent adverse events [28]

The study also highlights that LLMs still have an error rate in areas such as understanding complex questions or repeating answers in different interactions. Despite this, the lower error rate in advanced models such as GPT-4o (compared to GPT-3.5 and Copilot) indicates significant progress in reducing errors and understanding specific contexts. This suggests that as LLMs continue to evolve and optimize their responses, they could become a central tool in the training of informal caregivers [10].

### Practical Implications

LLMs represent a promising innovation in informal caregiver training. Their ability to provide accurate, accessible instructions at a low cost, combined with their adaptability to different scenarios, and personalized information makes them valuable tools for improving safety in home care.

Implementing LLMs in informal care settings could reduce home care errors and alleviate health care system burdens by minimizing preventable incidents. In addition, they can provide greater reassurance to caregivers and help ease their emotional burden. However, further research and enhancements are required for these models to achieve professional-level reliability.

Evaluation models for chronic care approaches (such as the Chronic Care Model [29] and IEMAC [30]) should revise their standards to incorporate the impact of LLMs) considering their growing contributions to supporting both clinical professionals and formal caregivers in performing their roles more effectively, as well as enabling informal caregivers to assume their responsibilities in-home care with greater autonomy.

### Future Research

Rapid technological advancements suggest the potential for incorporating visual aids, such as videos and diagrams, into LLM outputs to improve comprehension and execution of complex caregiving tasks. Future research should explore the utility, suitability, and acceptability of these developments among caregivers and professionals.

Research should also focus on (1) enhancing prompt design to improve the reliability of LLM responses and reduce errors; (2) evaluating the ability of LLMs to provide culturally sensitive caregiving instructions tailored to diverse practices; (3) assessing the long-term impact of LLM use on caregiver performance, patient safety, and caregiver well-being in real-world settings; and (4) addressing regulatory and ethical challenges, particularly those related to privacy, accountability, and overreliance on AI in caregiving contexts.

### Strengths

This study addresses the novel and underexplored area of using LLMs to train informal caregivers, emphasizing their potential to support nonprofessional care in home settings. By evaluating 3 widely used LLMs (GPT-3.5, GPT-4o, and Copilot), the study provides a robust comparison of their relative performance. The critical role of LLMs in reducing errors and improving patient safety in home care aligns with key healthcare priorities.

### Limitations

The findings may not be fully generalizable due to the selection of specific LLMs and caregiving scenarios, as well as the controlled study environment. Only 10 caregiving scenarios were analyzed, limiting the representation of the diversity of real-world home care situations.

LLM performance heavily depends on the clarity and specificity of prompts, which informal caregivers may find challenging to generate. In addition, the study focused on textual guidance, overlooking the potential of multimedia or interactive elements that could further enhance training outcomes.

A significant observation is that GPT models have, in certain contexts, outperformed doctors in patient interactions. This discrepancy may stem from the abundance and consistency of publicly available data used to train LLMs in patient-facing scenarios. However, the specificity and complexity of clinical decision-making require specialized datasets and targeted training, which remain underdeveloped for informal caregiving applications.

Another limitation lies in the lack of classification of errors based on their potential severity. While the analysis identifies omissions and other shortcomings in LLM-generated responses, it does not distinguish between errors that may cause minor inconveniences and those that could provoke severe patient safety risks. For instance, an omitted step in a procedure like insulin administration could have life-threatening consequences, whereas a similar error in routine hygiene tasks might only lead to mild discomfort. Future research should prioritize developing a framework to classify errors by severity to provide clearer insights into the risks associated with LLM use.

LLMs were evaluated against a theoretical gold standard, which represents an idealized set of instructions based on expert consensus. This approach does not fully account for the variability and practical constraints present in real-life decisions made by health care professionals. As a result, the differences between LLM performance and the gold standard may exaggerate the perceived gaps between LLMs and current practices, potentially underestimating the usefulness of LLMs in practical caregiving scenarios.

Finally, while the study underscores the potential of LLMs, their adoption requires a cautious and measured approach. Professional oversight is indispensable, especially in high-stakes contexts where even small errors could jeopardize patient safety. Ensuring adherence to evidence-based practices remains paramount. LLMs offer valuable support by enabling health care professionals to stay informed about the latest developments, enhancing their ability to provide tailored guidance for informal caregivers. However, until LLMs reach a level of reliability comparable to that of professionals, they should be regarded as complementary tools that augment human expertise rather than replace it.

### Conclusion

The integration of LLMs into informal caregiving holds significant promise for reducing errors in-home care and easing the strain on health care systems by preventing avoidable incidents. By complementing professional guidance and offering real-time support, these tools could revolutionize how informal caregivers manage their responsibilities. However, until their guidance is proven to be fully evidence-based and capable of minimizing errors to below human levels, the use of LLMs must be supervised by health care professionals equipped to interpret and apply the latest research evidence effectively.

In sum, further research and refinement are essential to improve the accuracy and reliability of these models, enabling them to meet professional standards.

## Appendix

Multimedia Appendix 1 Evaluation rubric.

Multimedia Appendix 2 Standardized prompts used.

## Abbreviations

AI: artificial intelligence
LLM: large language model

## Data Availability

The datasets generated or analyzed during this study are available from the corresponding author on reasonable request.
