# Supplementary material for: AI in Home Care—Evaluation of Large Language Models for Future Training of Informal Caregivers: Observational Comparative Case Study
Source: J Med Internet Res. 2025 Apr 28;27:e70703. doi: 10.2196/70703 (PMC12070015; doi:10.2196/70703)
Supplement: Multimedia Appendix 1 [file jmir_v27i1e70703_app1.docx]

Supplementary material. Evaluation rubric

**EVALUATION QUESTIONS**

**SPECIFICITY**
How specific do you consider the explanation generated by LLM model in response to the given situation? Please select a number from 1 to 5, where:
1 = Not specific at all
2 = Slightly specific
3 = Moderately specific
4 = Quite specific
5 = Completely specific

**CLARITY**
How well do you think you understood the explanation provided by LLM model regarding the given situation, in order to apply it in real life? Please select a number from 1 to 5, where:
1 = I did not understand the explanation at all
2 = I understood the explanation a little
3 = I partially understood the explanation
4 = I understood the explanation quite well
5 = I completely understood the explanation

**CONTEXT**
How would you rate the accuracy with which LLM model requested details about the condition of the given situation? (e.g., in the "Port-a-Cath" situation, did LLM model ask if the area where the device is inserted appears reddish, black, or normal?) Please select a number from 1 to 5, where:
1 = Did not ask for any specifications
2 = Asked for very few specifications
3 = Asked for some specifications
4 = Asked for several specifications
5 = Asked for all necessary specifications

**VARIABILITY ACCORDING TO CIRCUMSTANCES**
How would you rate LLM model´s explanation in terms of variability according to circumstances? (e.g., in the "Insulin" situation, the explanation may vary depending on the catheter being used to administer insulin). Please select a number from 1 to 5, where:
1 = Did not mention any possible variations
2 = Mentioned very few possible variations
3 = Mentioned some possible variations
4 = Mentioned several possible variations
5 = Mentioned all possible variations

**SELF-EFFICACY**
How confident do you feel about solving the situation you presented to LLM model after receiving its explanation? Please select a number from 1 to 5, where:
1 = Not confident at all
2 = Slightly confident
3 = Neither confident nor unconfident
4 = Quite confident
5 = Completely confident

**NUMBER OF ERRORS**
How many errors did you encounter in your interaction with LLM model? Please select a number from 1 to 5, where:
1 = More than 10 errors
2 = Between 6 and 9 errors
3 = 5 errors
4 = Between 1 and 4 errors
5 = No error

**TYPE OF ERRORS**
VII. What types of errors (A-D) have you identified in your interaction with LLM model?
A) LLM model omitted relevant information
B) LLM model provided more information than necessary or unrelated information
C) LLM model did not understand the question posed
D) LLM model repeated the same response to two different questions

Answer “Yes” or “No” depending on whether you have identified each type of error.
Provide qualitative examples if possible (e.g., When asked for handwashing instructions, it omitted the step of applying soap).

**COMPARISON QUESTIONS**

**SPECIFICITY/LEVEL OF DETAIL**
I. How would you rate the explanation provided by LLM model compared to the one in the Gold Standard? Please select a number from 1 to 5, where:
1 = Not specific or detailed at all compared to Gold Standard

2 = Slightly specific and detailed compared to Gold Standard

3 = Moderately specific and detailed compared to Gold Standard
4 = Quite specific and detailed compared to Gold Standard
5 = Very specific and detailed compared to Gold Standard

**RECOMMENDATIONS/ADVICE**
II. How would you rate LLM model’s explanation in terms of including reminders or advice like those found in Gold Standard? (e.g., Remember to keep the medicine cabinet out of reach of children or elderly people). Please select a number from 1 to 5, where:
1 = Did not give any reminders/advice
2 = Gave very few reminders/advice
3 = Gave some reminders/advice
4 = Gave several reminders/advice
5 = Gave all the necessary reminders/advice

**JUSTIFICATION**
III. How would you rate LLM model’s explanation in terms of justifying why a particular action should be taken, as done in Gold Standard? Please select a number from 1 to 5, where:
1 = I did not receive any explanation
2 = I received very little explanation
3 = I received some explanation
4 = I received quite a bit of explanation
5 = I received all the necessary explanation

**CONTENT**
IV. How would you rate LLM model’s explanation regarding mentioning the necessary steps? Please select a number from 1 to 5, where:
1 = Mentioned far fewer steps than those in Gold Standard
2 = Mentioned fewer steps than those in Gold Standard
3 = Mentioned the same steps as in Gold Standard

4 = Mentioned more steps than those in Gold Standard

5 = Mentioned far more steps than those in Gold Standard

**OVERALL EVALUATION**
V. After finishing the conversation with LLM model and reading the Gold Standard for each situation, how would you rate the explanation you received from LLM model compared to the one in the Gold Standard? Please select a number from 1 to 5, where:
1 = Much worse than the Gold Standard
2 = Worse than the Gold Standard
3 = Neither better nor worse than the Gold Standard
4 = Better than the Gold Standard
5 = Much better than the Gold Standard
