# Supplementary material for: AI in Home Care—Evaluation of Large Language Models for Future Training of Informal Caregivers: Observational Comparative Case Study
Source: J Med Internet Res. 2025 Apr 28;27:e70703. doi: 10.2196/70703 (PMC12070015; doi:10.2196/70703)
Supplement: Multimedia Appendix 2 [file jmir_v27i1e70703_app2.docx]

Supplementary material. Prompts

**INSULIN:**

Considering that I am an informal caregiver, explain the process of how to inject subcutaneous drugs such as insulin and glucagon (for diabetes) and heparins (for anticoagulation) as detailed as possible. This explanation must include all the necessary steps for the caregiver to perform the procedure safely and without risks for the person being cared for.

**PORT-A-CATH:**

Considering that I am an informal caregiver, explain the procedure for cleaning and maintaining a Port-a-Cath at home as detailed as possible. This explanation must include all the necessary steps for the caregiver to perform the procedure safely and without risks for the person being cared for.

**DIAPER CHANGES:**

Considering that I am an informal caregiver, explain the process of how to change a diaper as detailed as possible. This explanation must include all the necessary steps for the caregiver to perform the procedure safely and without risks for the person being cared for.

**TRANSFERS:**

Considering that I am an informal caregiver, explain the process of how to transfer the person being cared for from the bed to a wheelchair as detailed as possible. This explanation must include all the necessary steps for the caregiver to perform the procedure safely and without risks for the person being cared for.

**HEART FAILURE:**

Considering that I am an informal caregiver, explain the potential mistakes in caring for patients with heart failure at home, keeping in mind that the goal is to provide the best care and avoid errors that could affect the patient’s quality of life and disease progression. This explanation must include all the necessary information for the caregiver to perform their care tasks safely and without risks for the person being cared for.

Take into account that caring for a patient with heart failure involves not only managing medication and attending appointments and follow-ups but also monitoring symptoms to detect worsening conditions, tracking the patient’s weight and blood pressure, ensuring a diet low in salt and fat, and promoting physical exercise. Therefore, you should also explain the following points as clearly as possible:

1. Pharmacological treatment.
2. Monitoring warning symptoms, weight, and blood pressure.
3. Lifestyle care: diet and exercise.

**BLOOD PRESSURE:**

Considering that I am an informal caregiver, explain the process of how to monitor blood pressure as detailed as possible. This explanation must include all the necessary steps for the caregiver to perform the procedure safely and without risks for the person being cared for. It must also include the following topics:

1. What blood pressure is and normal values.
2. Measurement devices.
3. Simulation of measurement**.**

**CHOKING MANEUVERS:**

Considering that I am an informal caregiver, explain the process, in the most detailed way possible, to perform a maneuver to prevent choking in a person who is choking. This explanation must include all the necessary steps for the caregiver to perform the procedure safely and without risks for the person being cared for.

**CAREGIVER SELF-CARE:**

Considering that I am an informal caregiver, explain in as much detail as possible how to prevent injuries from poor posture or improper execution of movements, as well as exercises to stretch the neck and cervical spine, shoulders, and forearms. Keep in mind that this explanation is about the caregiver, meaning it is focused on informal caregivers taking care of themselves. Provide a total of 8 exercises.

**PRESSURE ULCERS:**

Considering that I am an informal caregiver, explain how to learn the strategies available to prevent or control the development of pressure ulcers as detailed as possible. This explanation must include all the necessary steps for the caregiver to perform the procedure safely and without risks for the person being cared for.

**HAND HYGIENE:**

Considering that I am an informal caregiver, explain the process of how to properly wash hands as detailed as possible. This explanation must include all the necessary steps for the caregiver to perform subsequent tasks ensuring their hands are as sterilized as possible.
